# The protective effect of the experimental TiF_4_ and chitosan toothpaste on erosive tooth wear in vitro

**DOI:** 10.1038/s41598-022-11261-1

**Published:** 2022-04-30

**Authors:** Monique Malta Francese, Isabela Vieira Bolzan Gonçalves, Mariele Vertuan, Beatriz Martines de Souza, Ana Carolina Magalhães

**Affiliations:** Department of Biological Sciences, Bauru School of Dentistry, University of São Paulo, Bauru, SP Brazil

**Keywords:** Dentistry, Dental materials, Oral conditions, Preventive dentistry

## Abstract

This study evaluated the protective effect of TiF_4_ and chitosan toothpaste on erosive tooth wear (ETW) in vitro. Enamel and dentin samples were randomly assigned to toothpastes (n = 12): (G1) TiF_4_ (1400 ppm F^−^), (G2) 0.5% chitosan (75% deacetylation, 500 mPas), (G3) TiF_4_ (1400 ppm F^−^) plus 0.5% chitosan (75% deacetylation, 500 mPas), (G4) Placebo, (G5) Erosion Protection (Elmex-GABA, 1400 ppm F^−^). Twelve samples were only eroded. All samples were submitted to erosive pH cycles and G1 to G5 to abrasive challenges using toothpastes’ slurries plus 45 s of treatment, for 7 days. The final profile was overlaid to the baseline one for the ETW calculation (µm). The data were subjected to Kruskal–Wallis/Dunn tests. TiF_4_ toothpastes, regardless of the presence of chitosan, were able to significantly reduce ETW compared to placebo, while chitosan alone was similar to placebo for both tissues. The toothpastes containing TiF_4_ were even superior to the commercial Elmex toothpaste on enamel, while they were similar on dentin; both were also significantly different from placebo for both tissues. TiF_4_ and Elmex toothpastes minimized the impact of brushing on eroded surface. In conclusion, TiF_4_ toothpastes, regardless the presence of chitosan, showed to be effective in minimizing ETW in vitro.

## Introduction

Erosive tooth wear (ETW) is the cumulative superficial loss of mineralized tooth substance due to chemical (erosion) and physical process (attrition, abrasion), in which erosion is the predominant etiological factor, associated with mechanical action of toothbrushing, for example^1,2^. The dentition naturally undergoes some wear overtime; however, the rate of wear should be extremely slow to maintain healthy the tooth morphology and functions throughout the lifetime^3^. Tooth wear can be defined as pathological if it is beyond the physiological level relative to the individual’s age, depending on its severity (dentin exposure) and if it interferes with the self-perception of well-being due to the presence of pain, function and/or aesthetics compromises^2,4^.

The increase in the prevalence and clinical detection of ETW in recent decades has attracted the attention of the dental community around the world^5–7^. The older age groups generally show high levels of wear^8^ and severe ETW may have impact on the quality of life of the affected individuals^9^.

Among the various strategies to control ETW, the most tested one is the application of fluorides^10–12^ especially those containing Sn^2+^ or Ti^4+^, polyvalent metals that interact with the tooth structure, forming a more acidic resistant layer compared to CaF_2_ induced by the application of conventional fluorides as NaF^11,13^. The daily application of a solution containing TiF_4_/NaF has promising results compared to those obtained with the commercial fluoridated solution (SnCl_2_ and NaF/AmF), clinically indicated for ETW, under in vitro and in situ models^13–15^. It has also shown to be more promising than a unique professional application of TiF_4_ varnish^16^. TiF_4_ incorporated into a toothpaste has also shown to reduce the ETW created by the association of erosive and abrasive challenges^17^.

Another compound of interest to control ETW is chitosan. Chitosan is a natural polymer derived from chitin deacetylation that has the ability to interact electrostatically with the tooth structure and easily adsorbs to enamel forming a protective layer^18^. Toothpaste containing chitosan (Chitodent®—Helmuth Focken Biotechnik) is able to inhibit ETW, showing similarity to that containing NaF; however, its protective effect is reduced when brushing forces are applied^19,20^.

To overcome this issue, chitosan has been added to fluoridated toothpastes^21^. Fluoridated toothpastes containing tin (around 3500 ppm Sn^2+^ and 1400 ppm F^−^), in the presence of chitosan (0.5%), have a better protective effect against erosive enamel wear than those with fluoride only^19,21^. Chitosan can increase the retention of tin to enamel, which may, at least in part, explain its protective effect in association with F^−^ and Sn^2+^^22^.

Taking this idea in mind, it is expected improvement of the protective effect of TiF_4_ toothpaste, already tested^17^, with the inclusion of chitosan into the formulae. Recently, we have shown that solutions containing TiF_4_/NaF and chitosan had a protective effect against enamel wear similar to the positive control (Elmex® GABA solution, containing Sn^2+^ and F^−^)^23^, while for dentin, no improvement in the effect of the fluoridated solution was seen with the addition of chitosan^24^.

Therefore, the aim of this work was to evaluate the protective effect of TiF_4_ and chitosan containing toothpaste on ETW on both enamel and dentin, in vitro. The null hypothesis is that no difference exists between TiF_4_ toothpaste, with or without chitosan, with respect to the protective effect on ETW.

## Materials and methods

### Sample preparation

The study was approved by the Local Ethics Committee for Animal Use (number 007/2020). Besides, all methods were performed in accordance with the relevant guidelines (ARRIVE guidelines) and regulations.

Seventy-two bovine enamel and 72 root dentin samples were prepared from incisors stored in 0.1% thymol solution (pH 7.0). The roots were separated from the crowns and both were then separately coupled to a prefabricated silicone mold (Biopdi, São Carlos, Brazil) and embedded in autopolymerizing acrylic resin, allowing the exposition of the labial surface. The samples were polished using silicon carbide sandpapers (320, 600 and 1200 grades of Al_2_O_3_ papers; Buehler, Lake Bluff, USA). Afterwards, the baseline profile was measured by using a contact profilometer and two thirds of the samples’ surfaces were protected with red nail polish (Risqué®, São Paulo, Brazil), to obtain two control areas^13,14^.

The samples were randomly assigned to 5 toothpastes (n = 12): (G1) experimental containing TiF_4_; (G2) chitosan; (G3) TiF_4_ plus chitosan; (G4) placebo (no F and no chitosan), (G5) commercial Erosion Protection (Elmex®—GABA, Switzerland). Twelve samples were only eroded (control). Table 1 shows the details about the toothpastes.

**Table 1:** Composition of the toothpastes under study (according to information available in the label) and their respective pH values (slurries’ pH, mean and S.D.).

Group	Toothpaste	Ingredients	Active ingredients	Fluoride content	pH
TiF_4_	Bauru fórmulas (Bauru, São Paulo, Brazil)	Carboxymethyl cellulose (CMC), glycerin, methylparaben, sorbitol, abrasive silica, titanium dioxide, cocobetaine, aqua qsp	Titanium tetrafluoride (TiF_4_)	1400 ppm F^−^	3.08 ± 0.09
Chitosan	Bauru fórmulas (Bauru, São Paulo, Brazil)	Carboxymethyl cellulose (CMC), glycerin, methylparaben, sorbitol, abrasive silica, titanium dioxide, cocobetaine, aqua qsp	0.5% Chitosan (75% deacetylation, 500 mPas)	Nothing	7.14 ± 0.20
TiF_4_ plus chitosan	Bauru fórmulas (Bauru, São Paulo, Brazil)	Carboxymethyl cellulose (CMC), glycerin, methylparaben, sorbitol, abrasive silica, titanium dioxide, cocobetaine, aqua qsp	Titanium tetrafluoride (TiF_4_) and 0.5% chitosan (75% deacetylation, 500 mPas)	1400 ppm F^−^	4.22 ± 0.03
Placebo	Bauru fórmulas (Bauru, São Paulo, Brazil)	Carboxymethyl cellulose (CMC), glycerin, methylparaben, sorbitol, abrasive silica, titanium dioxide, cocobetaine, aqua qsp	Nothing	Nothing	6.97 ± 0.23
Elmex® erosion protection	GABA International AG, Grabetsmattaweg, Switzerland	Glycerin, sorbitol, hydrated silica, aroma, cocamidopropyl betaína, sodium sacharine, hydroxyethylcellulose, aqua	Stannous chloride (SnCl_2_), amine fluoride (AmF), Sodium fluoride (NaF), 0.5% chitosan	1400 ppm F^−^	4.55 ± 0.07

The chitosan toothpaste was prepared as described for the experimental solution^23,24^. All toothpastes were diluted (1 part toothpaste to 3 parts deionized water by weight; hereafter named slurry) for the treatment. The pH of the slurries was measured in duplicate using a pH meter previously calibrated to pH 4.1 and 7.0 standards (Orion 3-star pH Bench Top, Thermo Electron Corporation, USA). The toothpastes were applied at their natural pH, since TiF_4_ is less effective in preventing tooth erosion when its pH is buffered to a high value^25^.

### Erosive and abrasive cycling

The samples were subjected to daily erosive and abrasive challenges for 7 days^26,27^. Erosion was induced 4 times a day by using 0.1% citric acid solution (pH 2.5) for 90 s (30 mL/sample) at 25 °C. The samples were then washed in deionized water (5 s) and immersed in artificial saliva^28^ (pH 6.8, 30 mL/sample) for 2 h between the erosive challenges at 25 °C.

After the first and the last daily erosive challenges, the samples from G1 to G5 were subjected to abrasion for 15 s (except the erosion only—G6), using toothbrushing machine (Biopdi®, São Carlos, Brazil), toothbrush (5460 ultrasoft Curaprox®, Kriens, Switzerland, 1 toothbrush/sample) and the toothpastes’ slurries (1:3 water, 15 mL/sample, 37 °C) under standardized velocity (3 linear movements/s) and force (1.5 N)^26,27^. Afterwards, the samples were kept for further 45 s in contact with the toothpastes’ slurries to complete 1 min of treatment and washed using deionized water for 10 s. The samples were kept in artificial saliva overnight completing 24 h of cycling.

After 7 days, the nail polish was removed by using acetone solution and the ETW (final profile) was measured.

### Contact profilometry

ETW was determined using a contact profilometer (Mahr Perthometer, Göttingen, Germany). Five equidistant surface scans of each sample were performed (4.12 ± 0.59 mm for enamel and 6.22 ± 1.20 mm for dentin, 250 μm apart from each other) at the baseline and at the final measurement. To achieve the repeatability, the samples presented an identification mark (small drillings made with drill ¼, Jet Carbide, Kerr, Joinvile, Brazil) and two scratches to delimitate the exposed area. They were inserted into a metal device (x and y axes determination, reproducibility of 0.08 μm), to allow the stylus to be accurately repositioned at each measurement. The baseline profile was compared to the final profile for the calculation of the ETW by using the software Marh Surf XCR20. This analysis was done under 100% humidity for dentin^13,14,23,24^.

The scans were superposed (final profiles versus baseline profile) and the average depth of the under-curve area was calculated, considering the limit of detection of the system of 0.5 μm^13,14,23,24^.

### Statistical analysis

The ETW data were statistically compared using Kruskal–Wallis followed by Dunn test, since no equality of variances was found (Bartlett’s test), for both tissues separately. The software applied was Graph Pad Instat (San Diego, USA) and the level of significance was set at 5%^13,14,23,24^.

### Ethics declarations

The study was approved by the Ethics Committee for Animal Use of Bauru School of Dentistry—USP (Number 007/2020). It follows the recommendations in the ARRIVE guidelines. All methods were performed in accordance with the relevant guidelines and regulations.

## Results

**Figure 1:**
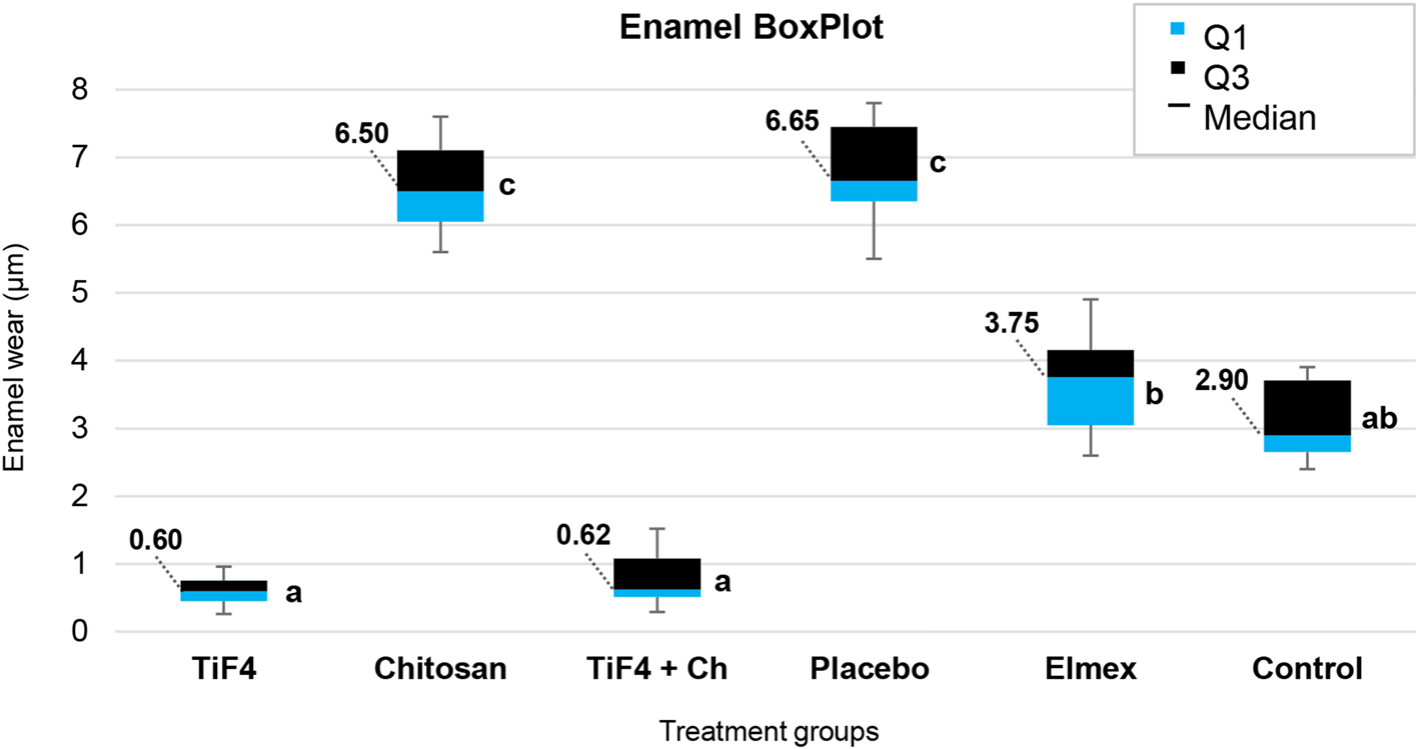
Box Plot of enamel wear (µm) after the experimental protocol according to each treatment group [median (interquartile range-II)]. *Q1* quartile1, *Q3* quartile 3. Kruskal–Wallis/Dunn test (p < 0.0001). Different letters show significant differences among the groups.

**Figure 2:**
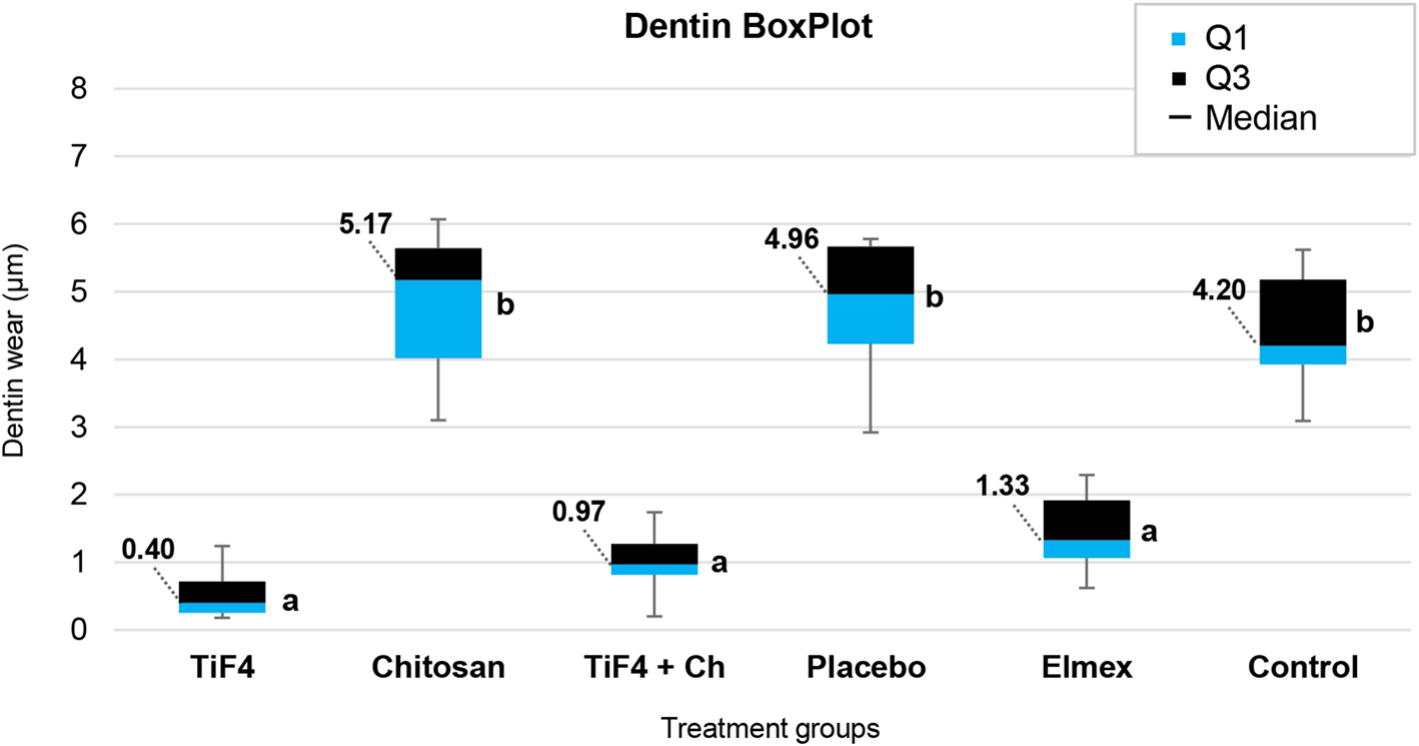
Box plot of dentin wear (µm) after the experimental protocol according to each treatment group [median (interquartile range-II)]. *Q1* quartile1, *Q3* quartile 3. Kruskal–Wallis/Dunn test (p < 0.0001). Different letters show significant differences among the groups.

The experimental TiF_4_ toothpastes, regardless of the presence of chitosan, were able to significantly reduce enamel and dentin wear (about 80% of prevention) compared to placebo, while chitosan alone was similar to placebo for both tissues. The toothpastes containing TiF_4_ were even superior to the commercial Elmex® toothpaste in reducing enamel wear, but they were similar in case of dentin, and both significantly reduced tooth wear compared to placebo (p < 0.0001). Both TiF_4_ and Elmex® toothpastes were not significantly different compared to the erosion condition for enamel and presented lower values compared to erosion for dentin, which means that they significantly reduced the abrasive brushing effect on eroded tooth surface (Figs. 1, 2).

## Discussion

The null hypothesis of this work was accepted, since the inclusion of chitosan did not improve the protective effect of TiF_4_ toothpaste, which in fact was even better than the positive control at least for reducing enamel wear. The experimental toothpastes containing TiF_4_ and chitosan demonstrated a protective effect of 89% for enamel and 78% for dentin compared to placebo, whereas the commercial Elmex® presented 43% and 71% of protective fraction, respectively.

According to previous studies, the protective capacity of TiF_4_ on the enamel is justified by the action not only of fluoride, but also of titanium^29,30^. TiF_4_ has been added to several formulations (such as varnish and solution), proving to be an effective compound against tooth demineralization (both caries and dental erosion) when compared to formulations containing NaF in vitro and in situ^13,15,30–32^ Titanium minimizes tooth demineralization, since it tends to complex with apatite, forming a “glaze” layer rich in titanium oxide and hydrated titanium phosphate, which is more acid-resistant than the layer of CaF_2_ induced by the application of NaF^30^. In addition, TiF_4_ induces greater CaF_2_ precipitation than NaF due to its low pH^30^.

Chitosan was added into the toothpaste containing TiF_4_, to improve its protective effect, since the literature has shown that the addition of chitosan to fluoridated solutions and toothpastes leads to a reduction in ETW^23,24,33,34^. A recent work has shown benefit of the association between TiF_4_/NaF and 0.5% chitosan on the protection of enamel wear in vitro^24^. This biopolymer is able to adsorb to enamel, creating a positively charged and more hydrophobic surface^20,35^, providing a mechanical barrier against acids^20^. This mechanism justifies its role as a mechanical barrier against the penetration of acids, contributing to the inhibition of demineralization^36^. However, when applied isolated (without fluoride), its protective effect is reduced by brushing forces, as shown by our study and others^19,20^. Our study also showed that the addition of chitosan to TiF_4_ toothpaste did not improve the protection, regardless the tooth substrate, indicating that chitosan, under the tested conditions, may not interact with the tooth surface producing an organic layer as expected.

An interesting result found in this study is that the experimental TiF_4_ toothpaste was superior to the commercial version indicated for controlling ETW in case of enamel. The commercial Elmex® Erosion Protection toothpaste has in its formulation: F (as AmF and NaF, 1400 ppm), Sn^2+^ (as SnCl_2,_ 3500 ppm), and chitosan (0.5%)^21,22,37^. Ganss et al.^22^ observed, in an in vitro study, applying more frequent erosive challenges and a longer treatment time with toothpaste, 68% reduction in enamel wear with the use of Elmex® Erosion Protection toothpaste compared to placebo. Schlueter et al.^21,37^, using similar methodology, but in situ, reported a reduction of approximately 50% of enamel wear by the use of Elmex® Erosion Protection compared to placebo, which is in agreement with our work.

Tin, like titanium, has an interaction with the tooth structure, being incorporated into the tooth surface and creating a mechanical barrier together with fluoride precipitates. When tin is combined with chitosan, there is a synergistic effect due to the formation of tightly connected multilayers^12,22,37^ acting as a shield for the deposition of Sn^2+^ and increasing the preventive effect of this complex structure against ETW. This scenario was not observed in the case of TiF_4_.

Differently from enamel, studies involving dentin are scarce. Elmex® Erosion Protection toothpaste reduces dentine wear, but not at superior level compared to a conventional fluoride toothpaste^38^. Tin may react with the dentin surface regardless of the presence of the demineralized collagen layer. In cases in which the organic matrix is preserved, phosphoproteins might attract the tin ion, which is then retained in the organic matrix to some extent but also accumulates in the underlying mineralized tissue. Under the absence of the demineralized organic matrix layer, the reaction is by precipitation^39^.

One factor that could have influenced the lack of synergic effect of chitosan and TiF_4_ is the erosive challenge, since the benefit of metal fluorides has been more evident when erosive challenges are longer^12,37^. Another important aspect is that the presence of abrasive silica in toothpaste can have limited the protective effect of chitosan associated with TiF_4_, when compared to fluoridated gels that do not have abrasive that could interact with chitosan^20,40^. It is also relevant to discuss that the effect of chitosan is also dependent of the low pH of the vehicle. In case of our study, the final pH value of toothpaste containing chitosan alone was close to neutral, which can reduce the protonation of the molecule and its protective effect, which should be considered. Previous works testing solution containing chitosan, at low pH, showed better effect on ETW^23,24^ than the tested toothpaste.

It is known that abrasive wear of eroded hard tissues is considered an adverse side effect of aggressive tooth brushing, which is determined mainly by the abrasiveness of toothpaste rather than by the toothbrush^41,42^. Based on this, the beneficial effect of TiF_4_ toothpaste, regardless of chitosan, may be not only due to the fluoride and titanium, but also to its low abrasivity. Thus, the need for further studies in the area is irrefutable, to analyze RDA/REA values of the experimental toothpastes and the effect of aggressive erosive challenges on their protection capacity. Other important point for the future is to buffer the toothpastes in order to have similar final pH (around 4.5) for all.

A limitation of the present study was the absence of human saliva, which is justified by the difficult to collect the amount needed for a pH cycling model of 7 days. Chitosan seems to have a great affinity to salivary proteins, reacting better with the tooth surface in the presence of those proteins^40,43,44^. Despite Luka et al.^45^ showed no improvement of the protective effect of Sn^2+^/F^−^/chitosan toothpastes under the presence of mucin *in* vitro, the present result shall be confirmed under in situ model, a condition closer to in vivo situation, with the presence of human saliva that may interplay the action of active compounds on the tooth^46,47^. Although chitosan did not have any protective effect when included into TiF_4_ toothpaste, it increased the toothpaste pH closer to the pH value of the commercial toothpaste, which is more suitable for a daily use.

## Conclusion

Based on the results, we conclude that TiF_4_ toothpastes, regardless of the presence of chitosan, are effective in minimizing tooth wear caused by brushing of eroded surface, which shall be confirmed under clinical studies. Although the chitosan was not able to improve the protective effect of the TiF_4_ toothpaste under this model, it increased the pH of the toothpaste to a more acceptable value for home-use oral care products.

## Data Availability

All data generated or analyzed during this study are included in this article.
